# Supplementary material for: How large and diverse are field populations of fungal plant pathogens? The case of Zymoseptoria tritici
Source: Evol Appl. 2022 Jul 15;15(9):1360–73. doi: 10.1111/eva.13434 (PMC9488677; doi:10.1111/eva.13434)
Supplement: Supplementary file 1 — Table S1 [file EVA-15-1360-s001.docx]

Table S1. Means over field assessments of STB incidence and STB severity measured at ten dates over ten consecutive years presented in Figure 1 of the main text (Suffert and Sache 2011; Suffert et al. 2018).

| Date | STB incidence | STB severity |
| --- | --- | --- |
| 2008-05-21 | 0.46 | 0.09 |
| 2009-05-27 | 0.42 | 0.08 |
| 2010-06-03 | 0.01 | 0.00 |
| 2011-04-28 | 0.27 | 0.03 |
| 2012-05-30 | 0.76 | 0.19 |
| 2013-05-22 | 0.33 | 0.19 |
| 2014-05-27 | 0.75 | 0.20 |
| 2015-06-04 | 0.97 | 0.21 |
| 2016-05-26 | 0.53 | 0.11 |
| 2017-05-22 | 0.24 | 0.03 |
